# Supplementary material for: Mechanisms Underlying Antipsychotic-Induced NAFLD and Iron Dysregulation: A Multi-Omic Approach
Source: Biomedicines. 2022 May 24;10(6):1225. doi: 10.3390/biomedicines10061225 (PMC9220331; doi:10.3390/biomedicines10061225)
Supplement: Supplementary file 1 [file biomedicines-10-01225-s001.zip › Table S1.pdf]

Supplemental Table S1: Binning Terms for NAFLD-Associated Pathways.

Pathway	Term	Rationale
NAFLD	Fatty Liver	Partial name description
NAFLD	Non-Alcoholic Fatty Liver Disease	Non-abbreviated name
NAFLD	Non-Alcoholic Fatty Liver Disease	Non-abbreviated name
Bile Pathways	Bile	Partial name description
Bile Pathways	Biliary	Potentially related pathologies
Bile Pathways	Choline Metabolism	Upstream pathway
Lipid Metabolism	Lipid	Partial name description
Lipid Metabolism	Fatty Acid	Alternate Name/Related Function
Inflammation	Edema	Alternate Name/Related Function
Inflammation	Toll	Upstream pathway
Inflammation	TLR	Upstream pathway
Inflammation	Chemokine	Upstream pathway
Inflammation	Cytokine	Upstream pathway
Serum Lipid Levels	Cholesterol	Alternate Name/Related Function
Serum Lipid Levels	HDL	Alternate Name/Related Function
Serum Lipid Levels	LDL	Alternate Name/Related Function
Serum Lipid Levels	VLDL	Alternate Name/Related Function
Serum Lipid Levels	Triglycerides	Alternate Name/Related Function
Obesity	Weight	Alternate Name/Related Function
Waist Circumference	Waist-to-Hip Ratio	Alternate Name/Related Function
Thrombosis	Embolism	Alternate Name/Related Function
Thrombosis	Embolus	Alternate Name/Related Function
Thrombosis	Clot	Alternate Name/Related Function
Thrombosis	Myocardial Infarction	Alternate Name/Related Function
Thrombosis	Stroke	Alternate Name/Related Function
