# Supplementary material for: Mechanisms Underlying Antipsychotic-Induced NAFLD and Iron Dysregulation: A Multi-Omic Approach
Source: Biomedicines. 2022 May 24;10(6):1225. doi: 10.3390/biomedicines10061225 (PMC9220331; doi:10.3390/biomedicines10061225)
Supplement: Supplementary file 1 [file biomedicines-10-01225-s001.zip › Table S2.pdf]

Supplemental Table S2: Binning Terms for Iron Homeostasis-Associated Pathways.

Pathway	Term	Rationale
Iron	Ferrous	Alternate Name/Related Function
Iron	Ferric	Alternate Name/Related Function
Anemia	Erythrocyte	Potentially related pathologies
Blood Values	Mean Corpuscular Volume	Specific Type
Blood Values	MCV	Specific Type
Blood Values	Hematocrit	Specific Type
Blood Values	HCT	Specific Type
Blood Values	Erythrocyte Indices	Specific Type
Blood Values	Red Blood Cell	Specific Type
Blood Values	RBC	Specific Type
Hemoglobin	Hgb	Abbreviation
Hemoglobin	Hb	Abbreviation
