# Supplementary material for: Mechanisms Underlying Antipsychotic-Induced NAFLD and Iron Dysregulation: A Multi-Omic Approach
Source: Biomedicines. 2022 May 24;10(6):1225. doi: 10.3390/biomedicines10061225 (PMC9220331; doi:10.3390/biomedicines10061225)
Supplement: Supplementary file 1 [file biomedicines-10-01225-s001.zip › Table S3-NAFLD Pathway Counts.pdf]

Supplemental Table S3: DE Traits from NAFLD-Associated Pathways[a].

Pathway	O_M	R_M	A_H	O_MR_M	O_MR_H	R_MR_H	O_MA_H	R_MA_H	R_HA_H[b]	$O_MR_MR_H$	$O_MR_MA_H$	$O_MR_HA_H$	$R_MR_HA_H$	$O_MR_MR_HA_H$
**NAFLD**	CYCS	MTTP<br>GPLD1		NDUFS3<br>NDUFB9<br>ADIPOQ	SDHD	IL-6			RAC1<br>HFE	NDUFS1<br>NDUFS3<br>NDUFS7<br>NDUFS8<br>NDUFB11<br>NDUFB2<br>NDUFB5<br>NDUFB7<br>NDUFB8<br>NDUFB9<br>COX1<br>COX8A<br>COX2<br>GSTP1		CYC1<br>UQCRC1<br>UQCRHL		Cacna2d1<br>NDUFA10<br>NDUFA11<br>NDUFA3<br>NDUFA5<br>NDUFA8<br>NDUFA9<br>NDUFA13
**Bile Synthesis, Secretion**	AQP1	GNAS<br>HSD3B7	PLA2G4A<br>SLC44A3		SLC44A5				DGKE<br>RAC1<br>PLPP1<br>PLPP2<br>RAC3		APOA1		DGKE<br>DGKZ	PDGFRB<br>PRKCA
**Cirrhosis**	CYCS	ROCK2<br>TFRC<br>MTTP<br>CXCL12	ELANE<br>CD209<br>MPO	ADIPOQ	IFNAR1<br>ACE	SMAD2<br>SMAD4<br>CCL2<br>COMT		LTA<br>TIMP1<br>TIMP2	ACVRL1<br>HFE	GSTP1				MMP11<br>MMP12<br>MMP14<br>MMP15

		CD14<br>GC<br>C4A				IL-6								MMP16<br>HLA-DQB1<br>HLA-A<br>HLA-C<br>PRKCA<br>ELN
**Chronic Hepatitis**	FADD<br>CYCS	MTTP<br>CXCL10	CLDN3<br>MPO		IFNAR1<br>ACE	CCL2<br>SMAD4		TIMP1<br>TIMP2	TICAM1<br>TICAM2	GSTP1<br>INPP5E	APOA1			MMP11<br>MMP12
**Pathway**	**O_M**	**R_M**	**A_H**	**O_MR_M**	**O_MR_H**	**R_MR_H**	**O_MA_H**	**R_MA_H**	**R_HA_H**	**$O_MR_MR_H$**	**$O_MR_MA_H$**	**$O_MR_HA_H$**	**$R_MR_HA_H$**	**$O_MR_MR_HA_H$**
**Chronic Hepatitis**		CXCL12<br>CD14<br>TFRC	NFATC2<br>CD209<br>STAT4			COMT<br>IL6		LTA	HFE	INPP5D				MMP14<br>MMP15<br>MMP16<br>PRKCA<br>HLA-A<br>HLA-C<br>HLA-DQB1
**Waist Circumference**	SERBP1	SEC14L2<br>IL1RAP<br>PLAUR	CLCA1<br>LPAR1<br>LY86<br>ZCCHC14			PSAT1<br>DAB2<br>IL-6<br>NUDT12				RBBP4<br>SEC23IP<br>RBBP5<br>MYL4		CCDC88C<br>HAS3<br>NPTN<br>TKT	CTSD	VAPA<br>CACNA2D3<br>HLA-DQB1<br>NDUFAF6
**Body Mass Index**	CMC1<br>SERBP1	MTTP<br>MTCH2	PLA2G4A<br>HHEX	ADIPOQ	CPT1B<br>L1CAM	PFKL<br>COMT		PABPC3<br>PPIF	SCARB2<br>SPTSSB	SEC23IP<br>GSTP1	APOA1			VAPA<br>PDGFRL

		CD14<br>GC<br>PPFIBP1<br>TRA2B<br>GNAS<br>QKI<br>C4A<br>FBN2<br>HPGD	GATA6<br>ITK<br>TMTC1<br>TMTC2		ACE<br>SLC44A5<br>GNAI1<br>PPP1R37	LMNA<br>DAB2<br>AP3D1<br>IL-6<br>NUDT12			TNS1<br>CD36<br>TUB<br>SH2B1<br>CCDC88C<br>HAS3<br>MTMR7<br>NEGR1<br>PLPP1<br>SEMA5A<br>SOCS2<br>UBE2E2<br>SOX11<br>PCGF3					PRKCQ<br>HLA-DQB1<br>CACNA2D1<br>CACNA2D3<br>MMP16
**Lipid Metabolism**	SGPL1<br>PGS1<br>GLB1	MTTP<br>PHOSPHO1<br>MECR<br>ACAA2	ETFDH<br>PLA2G4A<br>PSRC1<br>FABP1	ADIPOQ	AGPT3<br>ACE<br>CPT1B<br>CPT1C	LMNA<br>IL-6<br>PTPN11<br>CYP2J2	DECR1<br>DECR3	ALDH7A1<br>LTA	ST3GAL1<br>ST3GAL2<br>ST3GAL4<br>ST3GAL6<br>ACOT2		APOD<br>APOA1		DGKE<br>DGKZ<br>SLC27A1	PRKCQ<br>GLB1
**Pathway**	$\mathbf{O_M}$	$\mathbf{R_M}$	$\mathbf{A_H}$	$\mathbf{O_M R_M}$	$\mathbf{O_M R_H}$	$\mathbf{R_M R_H}$	$\mathbf{O_M A_H}$	$\mathbf{R_M A_H}$	$\mathbf{R_H A_H}$	$\mathbf{O_M R_M R_H}$	$\mathbf{O_M R_M A_H}$	$\mathbf{O_M R_H A_H}$	$\mathbf{R_M R_H A_H}$	$\mathbf{O_M R_M R_H A_H}$
**Lipid Metabolism**		ECI2			ECI1<br>DOCK7	LRP1			ST6GN3<br>ST6GN4<br>ST6GN6<br>RTN4					

									FUT2<br>HEXA<br>HEXB<br>MBOAT7<br>PCYT2<br>PLPP1<br>PLPP2					
**Serum Lipid Levels**	GP1BA<br>MTAP<br>PGS1<br>NEBL	MTTP<br>PPFIBP1<br>HMGCS2<br>CD14<br>ACAA2<br>HGPD<br>IL1RAP<br>IDI1<br>SLC12A4<br>QKI<br>C4A<br>HIBADH<br>ACAA2	FABP1<br>HNMT<br>LY86<br>MS4A4E<br>MID2<br>PSRC1<br>SNX31<br>TSHZ2<br>LARP7<br>UVRAG<br>CMIP<br>DEFA3<br>MPO<br>TMEM144<br>HHEX	ADIPOQ<br>SCFD1<br>SUMF1	ACE<br>CPT1B<br>DOCK6<br>DOCK7<br>MYBBP1A<br>PEPD<br>PRMT7	PTPN11<br>ACLY<br>CCL2<br>LRP1<br>COMT<br>LMNA<br>PFKL<br>DAB2<br>CYP51A1<br>NUDT7<br>IL-6<br>LAMC1<br>ZEB2	DECR1<br>DECR3	ALDH7A1<br>LTA	ST3GAL1<br>ST3GAL2<br>ST3GAL4<br>ST3GAL6<br>ACOT2<br>ST6GN3<br>ST6GN4<br>ST6GN6<br>DGKE<br>FUT2<br>HEXA<br>HEXB<br>MBOAT7<br>PCYT2<br>RTN4	SCFD1<br>RAB2A<br>SEC23IP<br>AGER<br>CHCHD3<br>G6PD<br>ITGA6<br>HNRNPD	APOD<br>APOA1		DGKE<br>DGKZ<br>SLC27A1	PRKCQ<br>GLB1

			PCNT<br>KCNK13<br>PLA2G4A						PLPP1<br>PLPP2					
**Obesity**	FGA	MTCH2<br>GNAS<br>FBN2	ANOS1<br>HHEX<br>SLC2A9	ADIPOQ	ACE<br>CPT1B<br>SH3BGRL	PTPN11<br>CCL2<br>SMAD2		LTA<br>PPIF<br>PABPC3	TUB<br>CD36<br>SH2B1	GSTP1<br>COX1<br>COX2	APOA1		CTSS<br>CAMK2B	PRRC2A<br>PRKCA<br>PRKCQ
**Pathway**	**O_M**	**R_M**	**A_H**	**O_MR_M**	**O_MR_H**	**R_MR_H**	**O_MA_H**	**R_MA_H**	**R_HA_H**	**$O_MR_MR_H$**	**$O_MR_MA_H$**	**$O_MR_HA_H$**	**$R_MR_HA_H$**	**$O_MR_MR_HA_H$**
**Obesity**		TCN2<br>TRA2B<br>GPLD1<br>HPGD	TMTC1<br>ZCCHC14<br>FABP1<br>MIF<br>STAT4<br>ITK<br>UVRAG<br>HNMT		PPP1R37<br>SLC44A5	SMAD4<br>TNFAIP1<br>CAST<br>COMT<br>IL6ST<br>LMNA<br>NOTCH2<br>IL-6<br>DAB2<br>CAMK2B<br>NUDT12			ST3GAL6<br>ACVRL1<br>ACVR2A<br>ACVR2B<br>NEGR1<br>HFE<br>RTN4					HLA-C<br>HLA-DQB<br>CACNA2D3
**Thrombosis**	GP1BA	KLK3	SLC44A3	MTHFD1	ACE	LRP1		LTA	HFE	SERPINC1	APOA1	CNTN1	CAMK2B	VAPA

	MTAP	KLKB1	ANOS1	ADIPOQ	F10	SMAD2		PPIF	GATA2	RBBP4		RIN2		ELN
	FGA	PLAUR	SELPLG	ATIC	PEPD	SMAD4		TIMP1	GATA3	AGER				HSPB8
	FXN	CXCL12	RNASE3	GSTO1	SRP19	COMT		TIMP2	ST3GAL1	G6PD				PRRC2A
		CD14	TMEM105			IL-6			TBC1D16	ITGA3				PTGES
		GNAS	ABCA9			PTPN11			UGGT2	ITGA6				PRKCQ
		NQO1	GATA6			TARDBP			IRAK1	PCCA				CACNA2D1
		GC	FABP1			AP3D1			TIGIT	GSTP1				CACNA2D3
		DOCK2	SERPINB8			CAMK2D			P2RY11	NDUFS7				MMP16
		PGLYRP2	SLC2A9			CYP2J2			CD36	RBBP5				PRKCI
		TCN2	MPO			NOTCH3			ACVRL1	MCCC2				PRKCZ
		QKI	TSHZ2			PSAT1			ACVRL2A					MYH9
		ACO1	ATOH8			SRSF1			ACVRL2B					HLA-DQB1
		FAM136A	ELANE			CCL2			IDH1					HLA-A
		FBN2	PSRC1			COMT			RBMS3					
		IL1RAP	TMEM105						SOX11					
		IGHG1	CMIP						TBC1D16					
		C4A	MIF						SEMA5A					
		C4B							LMO4					

**Inflammation**	FADD<br>CYCS	CD14<br>IL-12	SULT1A2<br>GATA6	ADIPOQ<br>MTHFD1	ACE<br>PGRMC2	CCL2<br>DAB2	DECR2	TIMP2<br>LTA	MTMR3<br>HFE	GSTP1<br>INPP5D	APOD<br>APOA1	RIN1<br>TRIM32	CTSD<br>CAMK2B	MMP11<br>MMP12
**Pathway**	**O_M**	**R_M**	**A_H**	**O_MR_M**	**O_MR_H**	**R_MR_H**	**O_MA_H**	**R_MA_H**	**R_HA_H**	**$O_MR_MR_H$**	**$O_MR_MA_H$**	**$O_MR_HA_H$**	**$R_MR_HA_H$**	**$O_MR_MR_HA_H$**
**Inflammation**	DECR2<br>APOD<br>ECM1<br>AQP1<br>FGA<br>FXN<br>GSTM2<br>NPC2<br>GP1BA<br>LUM<br>NEBL<br>RPIA<br>SAA1	IL-13<br>IL-16<br>IL-1A<br>IL-1B<br>IL-1RA<br>IL-1RAP<br>IL-27<br>IL-7<br>INFG<br>CXCL10<br>CXCL11<br>CXCL12<br>CXCL13<br>CXCL2<br>CXCL9<br>GC<br>MTTP	NLRP3<br>CRHBP<br>FOXP3<br>PLA2G4A<br>MPO<br>SELPLG<br>STAT4<br>BTK<br>ATOH8<br>DDT<br>C10ORF55<br>CLCA1<br>DACH1<br>FABP1<br>FOLR1<br>GDF15<br>HNMT	MASP1<br>GSTO1<br>TMPO<br>C9<br>ATIC	CPT1B<br>F10<br>POR<br>DOCK7<br>FLNA<br>LGALS1<br>HMGA1<br>P2RX4<br>SDHD<br>TOP2B<br>GNAI1<br>PPP1CA<br>PPP1CB<br>PPP1CC<br>IFNAR1<br>CPT1C<br>HMGA2	SMAD2<br>CAMK2B<br>PSAT1<br>IL-6<br>REEP6<br>LRP1<br>SMAD4<br>CAST<br>COMT<br>GJA1<br>IL6ST<br>LMNA<br>NOTCH2<br>PFKL<br>PTPN11<br>CAMK2D<br>CYP2J2			PTPN2<br>IRAK1<br>NECTIN2<br>LSS<br>NEGR1<br>NCAPH2<br>OSBP2<br>SCARB2<br>SEMA5A<br>SOCS2<br>ZFAND5<br>TUB<br>ABCA2<br>ABCB8<br>ATP8B2<br>CD36<br>ERI2	ITGA1<br>PCCA<br>SERPINC1<br>SERPIND1<br>RAP2B<br>RBBP4<br>RBBP7<br>AGER<br>G6PD			CAMK2D<br>ANXA2<br>CTSF<br>CTSG<br>CTSS<br>SLC27A1	MMP14<br>MMP16<br>MMP19<br>CACNA2D1<br>KIF5A<br>PRKCQ<br>PTGES<br>PRKCZ<br>PRKCA<br>MYH4<br>HLA-A<br>HLA-C<br>HLA-DQB1<br>HLA-DMA<br>EMLIN1<br>EMLIN2<br>ELN

		TCN2<br>HMGCS2<br>GNAS<br>NQO1<br>ROCK2<br>UGT1A6<br>DOCK2<br>C4A<br>C4B<br>FBN2<br>FTCD<br>HPGD<br>HYOU1<br>PLAUR<br>SLC12A4	NFATC2<br>PIGR<br>PSRC1<br>TSHZ2<br>SYK<br>MUC2<br>MIF<br>TMTC2<br>LPAR1<br>TYMP<br>TMTC1<br>ITK		PPP1R14B<br>BCL2L1	GNB2<br>CPSF4<br>NUDT22<br>NOTCH3<br>TIMM8A			FCHSD1<br>GATA2<br>WDR41<br>ACVRL1<br>ANKRD52<br>BSG<br>DLG4<br>FAM114A1<br>GRIN3A<br>GORASP1<br>HEXB<br>HGF<br>HAS3<br>RAC1<br>TICAM1					PDGFRB
**Pathway**	**O_M**	**R_M**	**A_H**	**O_MR_M**	**O_MR_H**	**R_MR_H**	**O_MA_H**	**R_MA_H**	**R_HA_H**	**$O_MR_MR_H$**	**$O_MR_MA_H$**	**$O_MR_HA_H$**	**$R_MR_HA_H$**	**$O_MR_MR_HA_H$**
**Inflammation**		TFRC<br>TMEM143<br>VCP							TICAM2					

[a]Drug/Host Abbreviations - O_M: OLAN/Mouse; R_M: RIS/Mouse; A_H: ARIP/Human Patients; R_H: RIS/Human Cells

[b]Sialyltransferase (ST6GALNAC) genes are abbreviated ST6GN
