# Supplementary material for: Mechanisms Underlying Antipsychotic-Induced NAFLD and Iron Dysregulation: A Multi-Omic Approach
Source: Biomedicines. 2022 May 24;10(6):1225. doi: 10.3390/biomedicines10061225 (PMC9220331; doi:10.3390/biomedicines10061225)
Supplement: Supplementary file 1 [file biomedicines-10-01225-s001.zip › Table S4-Iron Pathway Counts.pdf]

Supplemental Table S4: DE Traits from Iron Homeostasis-Associated Pathways[a].

Pathway	O_M	R_M	A_H	O_MR_M	O_MR_H	R_MR_H	O_MA_H	R_MA_H	R_HA_H[b]	$O_MR_MR_H$	$O_MR_MA_H$	$O_MR_HA_H$	$R_MR_HA_H$	$O_MR_MR_HA_H$
**Hemoglobin Levels**	SERBP1<br>SGPL1	TFRC	TSHZ2<br>HHAO<br>C1ORF162<br>LY86<br>SYNE2		RAB22A<br>F10<br>RNPS1<br>ACE<br>CHL1				JOSD2<br>SOX11<br>ACVR2A<br>FAM234A<br>HFE<br>HGF<br>HPCAL1<br>TFEC<br>SEMA5A	GSTP1<br>PCCA<br>RAB31<br>G6PD		CNTN1		PDGFRL<br>VAPA<br>HLA-A<br>HLA-C<br>HLA-DQB1
**Iron**		TRFC<br>ADHFE1<br>NBIA3	SYK<br>SLC44A3<br>TSHZ2	CLYBL<br>C9	SLC44A5	PSAT			PLXNA1<br>HFE<br>IBA57<br>LMO4					CACNA2D1<br>CACNA2D3<br>CACNA2D4<br>PRKCQ<br>HLA-A
**Iron Metabolism**		TFRC				FTH1			HFE<br>IB57					CACNA2D1<br>CACNA2D3<br>CACNA2D4<br>PRKCQ<br>HLA-A<br>HLA-C

														HLA-DQB1
**Hemochromatosis**		TFRC				SMAD 4 IL-6			HFE					HLA-A
**Anemia**	FGA GP1BA	TCN2 NQO1 TFRC	KCNK6 MIF MTHFD1		ACE AK1	IL6			GATA3 JOSD SOX11 FAM234A HFE HGF SESN3	G6PD GSTP1		RIN2		HLA-A HLA-C HLA-DQB1
**Pathway**	**O_M**	**R_M**	**A_H**	**O_MR_M**	**O_MR_H**	**R_MR_H**	**O_MA_H**	**R_MA_H**	**R_HA_H**	**$O_MR_MR_H$**	**$O_MR_MA_H$**	**$O_MR_HA_H$**	**$R_MR_HA_H$**	**$O_MR_MR_HA_H$**
**Anemia**									NEGR1 CD36 ZNF605 HLA-A					
**Thalassemia**					ACE				HFE	G6PD				HLA-C HLA-DQB1
**Blood Values[b]**		CCL1 QKI GC	TYMP TSHZ2 LY86		AK3		DECR1		GATA3 JOSD SOX11 FAM234A HFE HGF SESN3 NEGR1 ZNF605	SEC23IP PCCA	APOA1	RIN2		

[a]Drug/Host Abbreviations - O_M: OLAN/Mouse; R_M: RIS/Mouse; A_H: ARIP/Human Patients; R_H: RIS/Human Cells

[b]Included Blood Values – Hematocrit, Erythrocyte Count, Mean Corpuscular Volume, Erythrocyte Indices
